# Kindlin-1 Regulates Keratinocyte Electrotaxis

**DOI:** 10.1016/j.jid.2016.05.129

**Published:** 2016-11

**Authors:** Gaofeng Zhang, Yu Gu, Rumena Begum, Hongduo Chen, Xinghua Gao, John A. McGrath, Maddy Parsons, Bing Song

**Affiliations:** 1 Department of Dermatology, No. 1 Hospital of China Medical University, Shenyang, China; 2 School of Dentistry, College of Biomedical and Life Sciences, Cardiff University, Cardiff, UK; 3 Randall Division of Cell and Molecular Biophysics, Kings College London, London, UK; 4 St. Johns Institute of Dermatology, King’s College London, Guys Campus, London, UK

**Keywords:** EF, electric field, KS, Kindler syndrome, KS_MT, kindlin-1–mCherryW612A point mutation, KS_WT, Kindler syndrome cells infected with wild-type kindlin-1–mCherry, NHK, normal human keratinocyte, PH, pleckstrin homology, PI(3)K, phosphoinositide 3-kinase, PTEN, phosphatase and tensin homolog

## Abstract

Kindler syndrome (KS) is an autosomal recessive blistering skin disease resulting from pathogenic mutations in *FERMT1*. This gene encodes kindlin-1, a focal adhesion protein involved in activation of the integrin family of extracellular matrix receptors. Most cases of KS show a marked reduction or complete absence of the kindlin-1 protein in keratinocytes, resulting in defective cell adhesion and migration. Electric fields also act as intrinsic regulators of adhesion and migration in the skin, but the molecular mechanisms by which this occurs are poorly understood. Here we show that keratinocytes derived from KS patients are unable to undergo electrotaxis, and this defect is restored by overexpression of wild-type kindlin-1 but not a W612A mutation that prevents kindlin-integrin binding. Moreover, deletion of the pleckstrin homology domain of kindlin-1 also failed to rescue electrotaxis in KS cells, indicating that both integrin and lipid binding are required for this function. Kindlin-1 was also required for the maintenance of lamellipodial protrusions during electrotaxis via electric field-activated β1 integrin. Indeed, inhibition of β1 integrins also leads to loss of electrotaxis in keratinocytes. Our data suggest that loss of kindlin-1 function may therefore result in epithelial insensitivity to electric fields and contribute to KS disease pathology.

## Introduction

Kindlin-1 is predominantly expressed in epithelial tissues such as skin and intestine. Loss-of-function mutations in kindlin-1 causes Kindler syndrome (KS), which is characterized by skin blistering, fragility, and photosensitivity (Jobard et al., 2003, Kindler, 1954, Siegel et al., 2003). Keratinocytes from KS patients show defects in cell migration, adhesion, and proliferation (Has et al., 2009, Has et al., 2015, Lai-Cheong et al., 2009). In addition, KS keratinocytes show loss of polarized migration because of reduced cell adhesion and as a result of kindlin-1 dysfunction (Herz et al., 2006).

Kindlin-1 has been shown to bind to β1, β3, and β6 integrin cytoplasmic domains and enhance focal adhesion formation (Kloeker et al., 2004). The integrin family mediates cell adhesion to the underlying basement membrane that is critical for skin integrity. β1 integrins are the predominant receptor in basal keratinocyte focal adhesions and, through connections to the actin cytoskeleton, are key to controlling protrusion formation during cell migration (Cox et al., 2003, Saravanan et al., 2009). Physiological electric fields (EFs) are important to skin function and wound repair. Breaches of the epithelial layer generate an endogenous electric current, which is crucial in mediating cell migration, division, and polarization; angiogenesis; and nerve regeneration during wound healing (McCaig et al., 2005). Keratinocytes can sense and respond to physiological EFs and migrate specifically toward a cathode in vitro (Nishimura et al., 1996). However, the mechanisms controlling EF-induced directional migration of keratinocytes remain poorly understood. A recent report showed a role for β1 integrins in mediating fibroblast sensing and response to electric stimulation (Tsai et al., 2013). Extracellular calcium (Fang et al., 1998), EGFR (Pu et al., 2007), cAMP (Pullar and Isseroff, 2005), and the phosphoinositide 3-kinase (PI[3]K)–phosphatase and tensin homolog signaling pathway (Zhao et al., 2006) have also been implicated, but the receptor signals initiating EF-induced polarity are not defined.

In this study, we describe a mechanism by which kindlin-1 mediates efficient keratinocyte electrotaxis through activation of β1 integrins. Our data show that kindlin-1 regulates the distribution and maintenance of lamellipodial protrusions and integrin activation through association with phospholipids and that this is required for polarization and directed electrotaxis. These data provide the evidence that specific focal adhesion proteins are required for optimal cell sensing of electric gradients and further suggest that a potential defect in this pathway in KS patients may contribute to disease pathology.

## Results

### KS keratinocytes are defective in electrotaxis

**Figure 1:**
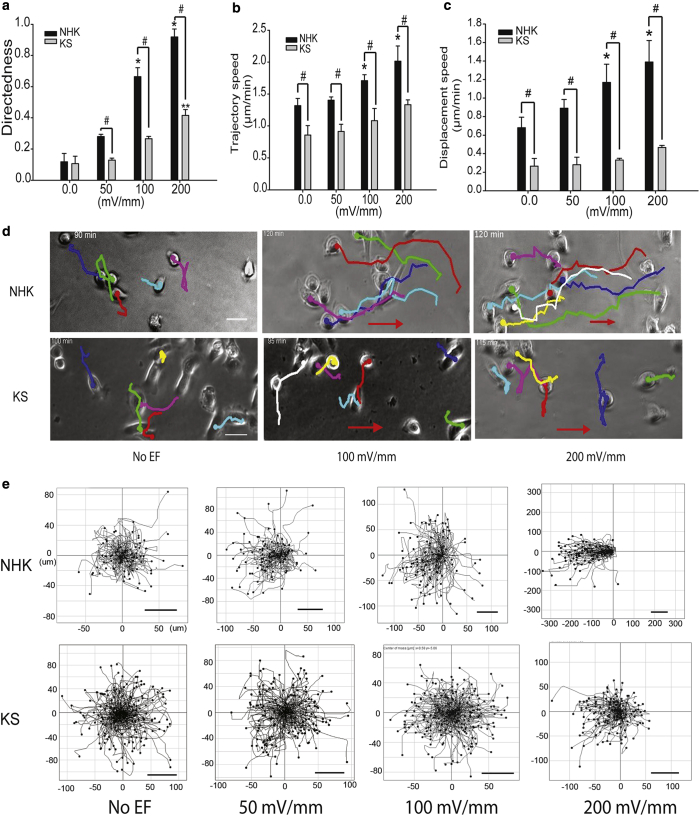
**Electrotaxis is impaired in KS cells.** (**a–c**) Graphs show (**a**) migration directedness, (**b**) trajectory speed, and (**c**) displacement speed of NHK and KS cells with EF-specified treatment from 2-hour movies. (**d**) Representative time-lapse images show movement of NHK (top panel) and KS (bottom panel) in response to indicated EF (see Supplementary Video S1). The track lines indicate migration paths. (**e**) The cell migration trajectories of approximately 200 cells of NHK (top panel) and KS (bottom panel) in EF ranges from 0 to 200 mV/mm are presented with starting positioned at origin (0,0); x- and y-axes give distance in micrometers. EF vector is horizontal, with cathode to the left. Results are presented as means ± standard error of the mean, n ≥ 3 experiments. ^∗^NHK (EF vs. no EF), ^∗∗^KS (EF vs. no EF), ^#^NHK vs. KS. *P* < 0.05. Scale bar = 100 μm. EF, electric field; KS, Kindler syndrome; NHK, normal human keratinocyte.

Kindlin-1 has previously been shown to be required for efficient migration of keratinocytes and loss or reduced levels of this protein in KS leads to defective adhesion assembly and migration (Herz et al., 2006). To determine whether kindlin-1 was also involved in response to EFs, a physiological EF range of 0–200 mV/mm were used to examine the abnormalities of KS keratinocytes compared with normal human keratinocytes (NHK). Migration directedness, trajectory, and displacement speed are used to describe cell responses to EF stimulation. Directedness indicates migration direction, where 0 is random migration and 1 is directional movement toward the cathode (Guo et al., 2010, Song et al., 2007). The trajectory and displacement speeds describe how fast cells migrate from the start to the endpoint. NHK exhibited dose-dependent responses in directedness, trajectory, and displacement speed to increasing EFs up to 200 mV/mm (Figure 1a–c). By contrast, KS cells did not show significant electrotactic responses to any conditions tested with the exception of 200 mV/mm EF (Figure 1a–c). No further increase in directional migration was observed over longer imaging periods (up to 4 hours; data not shown). Detailed analysis of the time-lapse movies showed that NHK responded immediately to EF stimulation and migrated directionally toward the cathode, whereas KS cells showed significantly lower electrotactic response (Figure 1d and e, and see Supplementary Video S1 online). Thus, we conclude that loss of kindlin-1 results in significantly impaired keratinocyte electrotaxis.

### Kindlin-1 mediates keratinocyte electrotaxis through binding to β1 integrins

**Figure 2:**
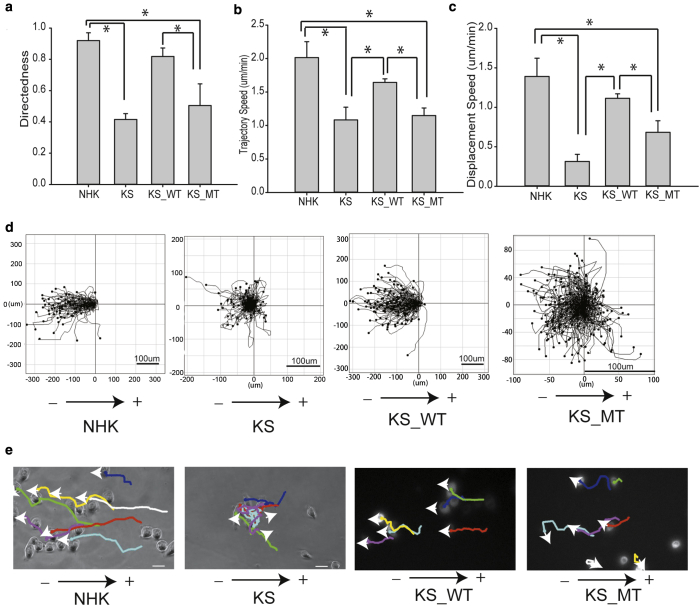
**Kindlin-1 mediates keratinocyte electrotaxis through binding to β1 integrins.** The (**a**) migration directedness, (**b**) trajectory speed, and (**c**) displacement speed of KS_WT (WT kindlin-1 re-expression in KS cells) and KS_MT (W612A kindlin-1 re-expression in KS cells). (**d**) The cell migration trajectories of approximately 150 NHK, KS, KS_WT, or KS_MT cells in 200 mV/mm are presented with starting position at origin (0,0), x- and y-axes give distance in micrometers. Arrows indicate the electric field direction, and arrowheads indicate the direction of cell migration. (**e**) Representative images of NHK, KS, KS_WT, and KS_MT from time-lapse sequences showing cell movement with 200 mV/mm (see Supplementary Video S2). EF vector is horizontal with cathode to the left; arrows indicate the EF direction. The track lines indicate migration paths, with arrowheads indicating migration direction. Results are presented as means ± standard error of the mean, n ≥ 3 experiments. ^*^P < 0.05. Scale bar = 100 μm. KS, Kindler syndrome; KS_MT, kindlin-1–mCherryW612A point mutation; KS_WT, Kindler syndrome cells infected with wild-type kindlin-1–mCherry; NHK, normal human keratinocyte.

Kindlin-1 has been shown to associate directly with integrin cytoplasmic domains and is required for full activation of β1 integrins and, subsequently, cell adhesion and migration (Herz et al., 2006, Lai-Cheong et al., 2009, McMillan et al., 2007). Recent studies have shown that the C-terminal F3 domain of kindlin-1 containing W612 is required for kindlin-integrin binding. A W612A point mutation in kindlin-1 blocks the binding of kindlin-1 to the tail of integrin β1, thus abolishing its ability to activate integrins (Harburger et al., 2009). To explore whether the deficient response of KS cells to EF is due to the loss of kindlin-1 binding to integrins, we stably infected KS cells with wild-type kindlin-1–mCherry (KS_WT) or kindlin-1–mCherryW612A point mutation (KS_MT) and analyzed their responses to EF. KS_WT cells showed rescue responses similar to those of NHK, suggesting that loss of kindlin-1 was responsible for the defects in KS cells (Figure 2a–c). However, KS_MT cells failed to show the rescue effects as KS_WT cells did, with the reduced electrotactic response in all cases (Figure 2a–c). Cell trajectories (Figure 2d) and time-lapse images (Figure 2e, and see Supplementary Video S2 online) confirmed these observations. These data suggest that kindlin-1 is required for EF-induced directional migration of keratinocytes, and interaction with β1 integrins is one of the requirements for EF sensing.

### Kindlin-1 is required for protrusion polarization in electrotaxis

**Figure 3:**
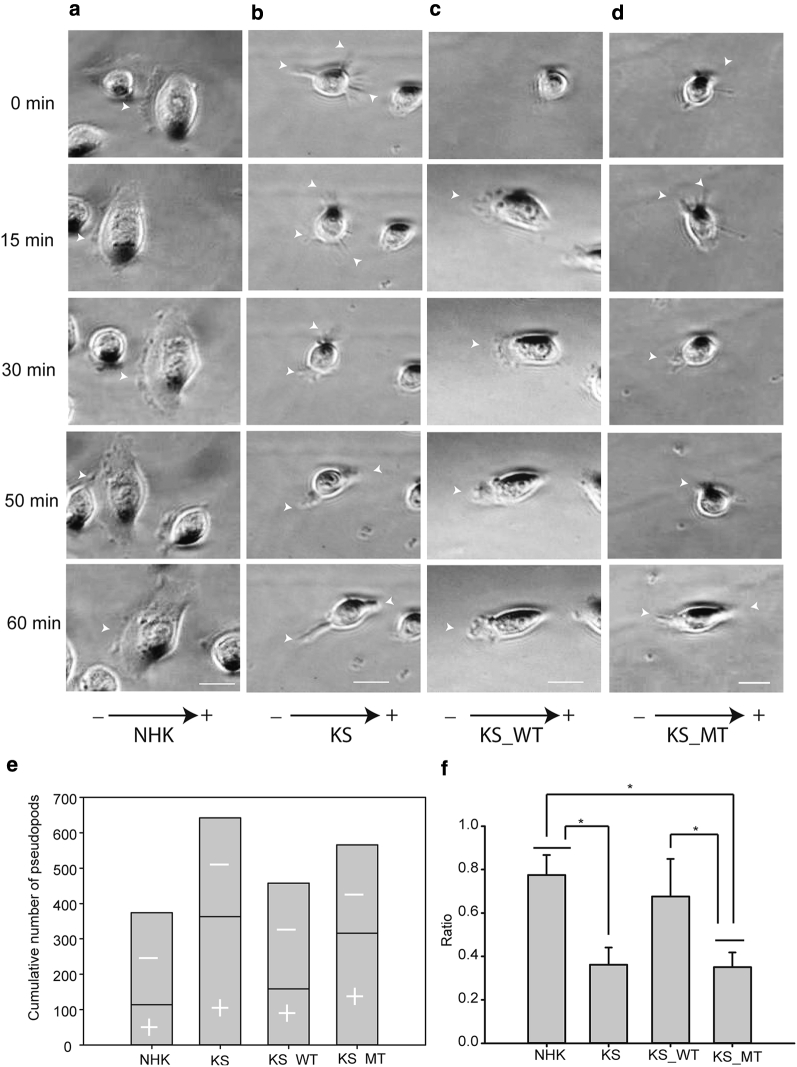
**Kindlin-1 is required for protrusion polarization in electrotaxis.** (**a–d**) Representative time-lapse images showing pseudopod formation and localization over 1 hour in EF (200 mV/mm) stimulation of (**a**) NHK, (**b**) KS, (**c**) KS_WT, and (**d**) KS_MT cells (see Supplementary Video S3). The EF vector is horizontal, with cathode to the left. The arrowheads indicate pseudopods. (**e**) Cumulative number of pseudopods of cells in **a**–**d** over EF stimulation. Upper rectangle indicates the number of cathode-directed pseudopods; lower rectangle indicates anode-directed pseudopods. (**f**) The ratio of cathodal pseudopods against all protrusions was analyzed. Results are presented as means ± standard error of the mean, n ≥ 3 experiments. ^∗^*P* < 0.05. Scale bar = 50 μm. min, minutes; KS, Kindler syndrome; KS_MT, kindlin-1–mCherryW612A point mutation; KS_WT, Kindler syndrome cells infected with wild-type kindlin-1–mCherry; NHK, normal human keratinocyte.

The formation of F-actin containing lamellipodia and filopodia is critical for directional migration and requires the interaction of external guidance cues, adhesion receptors, and cytoplasmic adaptors (Fukata et al., 2003, Petrie et al., 2009, Watanabe et al., 2005). Analysis of movies showed that during 2 hours of EF stimulation, NHK displayed a persistent lamellipodia formation toward the cathode (Figure 3a, and see Supplementary Video S3 online). However, KS cells formed filopodia-like protrusions in random directions, resulting in a random migration pattern (Figure 3b, and see Supplementary Video S3). KS_WT cells exhibited formation of a single polarized lamellipodia (Figure 3c, and see Supplementary Video S3), whereas KS_MT cells failed to form stable polarized protrusions similar to KS cells (Figure 3d, and see Supplementary Video S3). Similarly, KS_WT cells showed a ratio of cathode-facing protrusions similar to NHK, whereas KS_MT cells exhibited significantly lower cathode-facing protrusions (Figure 3f). These data show that kindlin-1–integrin binding is required for the formation of protrusions that lead to efficient keratinocyte electrotaxis.

### Kindlin-1 is important for the maintenance of EF-induced protrusions

**Figure 4:**
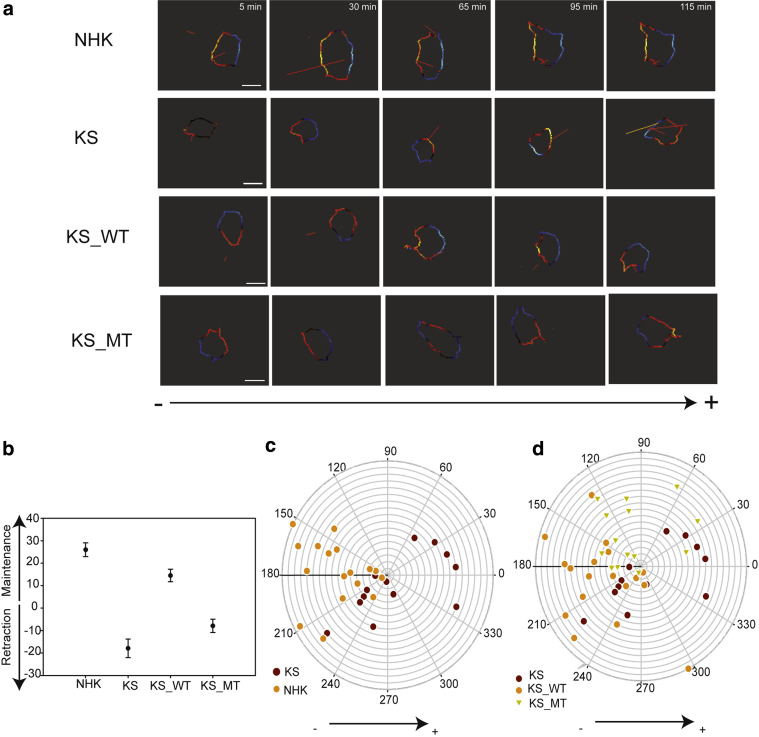
**Kindlin-1 is important for the maintenance and distribution of EF-induced protrusions.** Pseudopod maintenance and retraction during electrotaxis were analyzed using the Quimp algorithm. (**a**) Representative images show pseudopod dynamics in NHK, KS, KS_WT, and KS_MT treated with 200 mV/mm for 1 hour (see Supplementary Video S4). Red indicates protrusion maintenance, and blue indicates membrane retraction. (**b**) The maintenance and retraction scores of a pseudopod according to its contribution to cell body transition in previous and after image frame. The graph shows pseudopod scores of NHK, KS, KS_WT, and KS_MT in electrotaxis. Minus indicates retraction, and positive indicates maintenance. (**c, d**) Pseudopod distribution of (**c**) NHK and KS or (**d**) KS, KS_WT, and KS_MT cells. The EF vector is horizontal, with cathode to the left. Scale bar = 50 μm. KS, Kindler syndrome; KS_MT, kindlin-1–mCherryW612A point mutation; KS_WT, Kindler syndrome cells infected with wild-type kindlin-1–mCherry; NHK, normal human keratinocyte.

The higher probability and longer maintenance of protrusion in a certain direction, the higher the possibility that cells migrate in that direction persistently (Petrie et al., 2009). We used the Quimp pseudopod analysis algorithm to further explore the effects of kindlin-1 on pseudopod maintenance under EF stimulation (Bosgraaf and Van Haastert, 2010b). Cell boundaries were masked and assigned different colors according to their dynamic behavior (red indicated protrusion, and blue indicated retraction) (Figure 4a). NHK and KS_WT cells showed persistent maintenance of the pseudopod toward the cathode, whereas KS and KS_MT failed to do so, with short-lived pseudopod generation in random directions (Figure 4a, and see Supplementary Video S4 online). We further calculated the pseudopod maintenance between consecutive time points throughout the time-lapse sequence. To classify the morphological behavior, we defined cathodal protrusions as those occurring between 135° and 225° and anodal protrusion as between –45° and 45° with respect to the EF source, because these regions provide the most representative directions with respect to cathode or anode. Data showed that KS cells had a lower pseudopod maintenance score compared with NHK cells, and this was rescued in KS_WT but not KS_MT cells (Figure 4b). The summarized distribution of pseudopod maintenance (horizontal EF vector, cathode facing left; Figure 4c and d) showed the pseudopod of KS and KS_MT cells distributed randomly, whereas NHK and KS_WT cell protrusions were concentrated specifically in the region 135–225° facing toward the cathode (Figure 4c and d).

### Kindlin-1–mediated integrin β1 activation promotes keratinocyte electrotaxis

**Figure 5:**
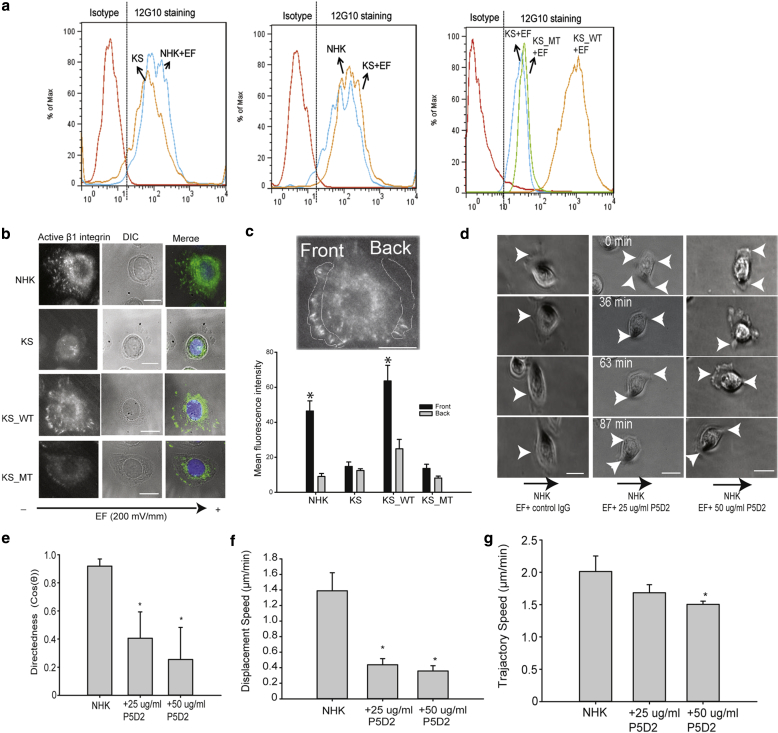
**Kindlin-1–mediated β1 integrin activation is required for keratinocyte electrotaxis.** (**a**) FACS shows an increase of peak fluorescent of active β1 integrin in (left) EF-treated NHK but not (middle) EF-treated KS; EF-treated KS_WT showed a much stronger fluorescent signal than (right) EF-treated KS and KS_WT cells. (**b**) Representative confocal images of active β1 integrin in NHK, KS, KS_WT, and KS_MT after EF. (**c**) Example image showing regions used for intensity analysis. Graph shows mean intensity values of active β1 integrin in the front versus rear of migrating cells. (**d**) NHK cells treated with indicated β1 integrin inhibition antibody P5D2 during EF (see Supplementary Video S5). Arrowhead indicates pseudopod. EF is horizontal, with cathode to the left. (**e**) Migration directedness, (**f**) displacement speed, and (**g**) trajectory speed of NHK with indicated β1 integrin inhibition antibody. Results are presented as means ± standard error of the mean, n ≥ 6. ^∗^*P* < 0.05. Scale bar = 50 μm. EF, electric field; KS, Kindler syndrome; KS_MT, kindlin-1–mCherryW612A point mutation; KS_WT, Kindler syndrome cells infected with wild-type kindlin-1–mCherry; Max, maximum; min, minutes NHK, normal human keratinocyte.

Integrins have been reported to be important in mediating EF-induced migration of keratinocytes (Pullar et al., 2006). To determine whether EF induces β1 integrin activation in a kindlin-1–dependent manner, FACS analysis was performed on all cell lines after EF stimulation by using the 12G10 antibody that specifically recognizes the active conformation of human β1 integrins (Mould et al., 1995). EF stimulation led to an increase in integrin activation in NHK and KS_WT cells but not in KS or KS_MT cells (Figure 5a). Immunostaining and intensity analysis of cells fixed immediately after exposure to EF gradients confirmed that NHK and KS_WT cells showed higher active integrin levels at focal adhesions polarized toward the EF cathode gradient that were not present in KS and KS_MT cells (Figure 5b and c). These results show that EF induces integrin activation in a kindlin-1–dependent manner.

To further verify that activation of β1 integrins is required for EF-induced directional migration, we assessed the electrotactic response of NHK cells pretreated with β1 integrin function-blocking antibody P5D2. Integrin inhibition in NHK resulted in the loss of persistent pseudopods facing the cathode (Figure 5c, and see Supplementary Video S5 online) and a dose-dependent reduction of electrotactic responses (Figure 5d–f), directedness, and displacement speeds (Figure 5d and e). However, trajectory speeds were reduced only when P5D2 was used at 50 μg/ml (Figure 5f). This altered response was further shown with circular graphs (see Supplementary Figure S1 online). These data confirm that β1 integrins are required for persistent protrusion and directional persistence in EF-induced keratinocyte migration.

### The kindlin-1 pleckstrin homology (PH) domain is required for electrotactic response in keratinocytes

**Figure 6:**
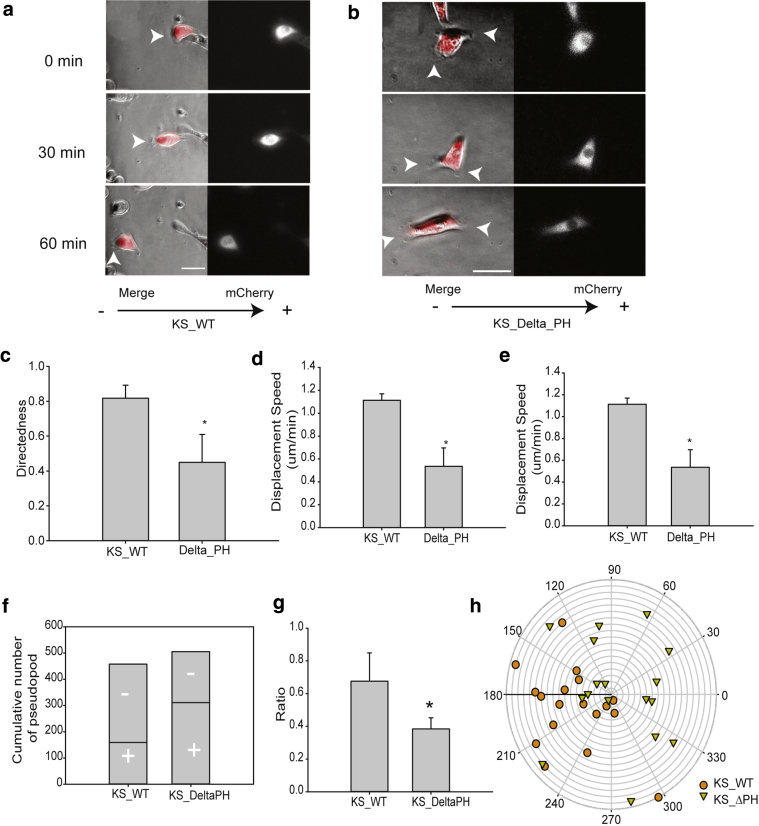
**The Kindlin-1 PH domain is required for electrotactic response of keratinocytes.** KS_WT and PH domain-deleted KS_Delta_PH cells were treated with 200 mV/mm for 1 hour. Representative time-lapse images show protrusion formation in (**a**) KS_WT and (**b**) KS_Delta_PH cells (see Supplementary Video S6). EF vector is horizontal, with cathode to the left. Arrowheads indicate protrusions. Graphs showing (**c**) directedness, (**d**) displacement speed, and (**e**) trajectory speed of cells as in **c**. The (**f**) cumulative number of pseudopods and ratio of (**g**) cathode-facing pseudopods and (**h**) pseudopod distribution are summarized. The – and + indicate cathode and anode, respectively. Results are presented as means ± standard error of the mean, n ≥ 3 experiments. ^∗^*P* < 0.05. Scale bar = 100 μm. KS_WT, Kindler syndrome cells infected with wild-type kindlin-1–mCherry; min, minute; PH, pleckstrin homology.

PI(3)K and downstream effector PIP3 have been reported to control electrotactic responses of several cell types (Liu et al., 2015, Zhao et al., 2006). Kindlin-1 contains a PH domain within the F2 subdomain, and this site has been proposed to bind both PIP2/PIP3 phospholipids (Ali and Khan, 2014, Liu et al., 2011). To investigate whether phospholipid binding was required for the kindin-1 dependent EF responses, KS cells were stably transduced with kindlin-1 lacking the PH domain (delta-PH). Analysis showed that deletion of the PH domain resulted in a diminished response to EF (Figure 6a and b, and see Supplementary Video S6 online). The directedness, trajectory displacement speed, and cathode-facing protrusions were also significantly reduced compared with KS_WT cells (Figure 6c–h). This indicates that phospholipid binding is required for kindlin-1 and integrin-dependent EF-induced directional migration in keratinocytes.

## Discussion

In this study, we showed a role for kindlin-1 in regulating pseudopod protrusion and maintaining directed cell migration in electrotaxis. We report that KS cells are defective in EF-mediated directional migration because of loss of function of kindlin-1. Previous work has shown that kindlin-1 is required for keratinocyte adhesion and spreading, and we show that kindlin-1 acts to maintain EF-induced asymmetric redistribution of pseudopod protrusions and polarized β1 integrin activation, which is in turn required for cathodal migration in response to EF. Our findings that re-expression of wild-type, but not W612A-kindlin-1, rescued the deficient electrotactic response of KS cells, strongly implies that kindlin-integrin association is required for efficient keratinocyte electrotaxis. Phospholipid signaling is a potential upstream regulator of kindlin-1–regulated electrotaxis, because we also showed that kindlin-1 lacking the PH domain failed to rescue the electrotactic response in KS cells. Our study reveals the role of kindlin-1 in mediating the directed migration response of keratinocytes to EFs and suggests that the membrane environment may be crucial to establishing an adhesion-dependent polarized guidance cue in response to EF stimulation.

Skin wounds generate endogenous EFs ranging from 70 to 200 mV/mm/ with variation across different species of animals and humans (Nishimura et al., 1996, Nuccitelli et al., 2011, Nuccitelli et al., 2008). The wound induces an EF gradient with the cathode at the wound center and the anode at the surrounding region. The transepithelial potential difference generated by epithelial ion transportation drives lateral ion current flow through epithelium because of the loss of transepithelial potential difference in wound site, and this is maintained until wound closure. This wound-induced physiological electric current is an early signal for keratinocytes to migrate toward the wound center (Nishimura et al., 1996). Physiological EFs also induce human keratinocyte directional migration to the cathode in vitro (Sheridan et al., 1996). The velocity of keratinocytes in response to EFs starts to increase from 50 mV/mm to 200 mV/mm (Nishimura et al., 1996). Extracellular matrix has been reported as an enhancer of the electrotactic response, and the hierarchy of response is reported to be collagen I and IV or plastic > fibronectin > laminin. Thus, the cell responses to EF ranges chosen in our experiments (0–200 mV/mm) on plastic supports are consistent with these previous reports.

It is still not clear how cells sense EF and how signals transduced to the cytoskeleton subsequently promote directional migration of the cells. Inhibitors of protein kinase C, Ca^2+^/calmodulin-dependent protein kinase, and myosin light chain kinase do not reduce either migration velocity or directedness significantly; however, both protein kinase A and PI(3)K inhibitors can effectively reduce cell responses to EF (Pullar and Isseroff, 2005, Zhao et al., 2006). This implies that there are divergent upstream signaling pathways regulating cell motility and sustained cell response to EF. Chemotaxis receptors are responsible for sensing the chemoattractant gradient and initiating directional migration. Equivalent receptors for electrotaxis have not yet been clearly identified. Directionality requires a degree of motility, and some molecules are required for both directional sensing and motility. Similarly, in cases where loss of a molecule results in total loss of motility, directional persistence becomes a meaningless parameter to analyze. Loss of kindlin-1 does not inhibit motility under basal conditions, suggesting that loss of migration speed alone cannot account for the loss of response to EF.

A commonly proposed mechanism for directional migration of a cell in response to a variety of stimuli is to initiate sustained cell polarization (Huttenlocher, 2005). This can be accomplished through the formation of front protrusion and subsequent rear retraction through extracellular signal relaying to cytoskeletal signaling molecules. Keratinocytes express both kindlin-1 and kindlin-2, which share some roles in adhesion formation and actin cytoskeleton organization (He et al., 2011). The perinatal lethality of kindlin-1 knockout mice and defects in KS patients caused by kindlin-1 null mutations confirms the importance of kindlin-1 in epithelial tissue development and homeostasis (Lai-Cheong et al., 2008, Ussar et al., 2008). Moreover, kindlin-1 functions independently of kindlin-2, because loss of kindlin-1 expression does not affect kindlin-2 expression or function (Bandyopadhyay et al., 2012, Lai-Cheong et al., 2008, Rognoni et al., 2014). The small guanosine triphosphatases are pivotal molecules in mediating actin rearrangement and protrusion formation; Cdc42 and Rac have been shown to be required for EF-induced growth cone turning toward the cathode in neurons (Rajnicek et al., 2006). In KS cells, guanosine triphosphate-bound, active Rac and RhoA are diminished (Has et al., 2009), and our data shows that KS cells form small filopodia-like protrusions rather than the larger directed lamellipodia seen in NHK. It is therefore plausible that this is due to defective regulation of RhoA/Rac reciprocity that is required for efficient protrusion formation (Machacek et al., 2009).

Integrins link cells to extracellular ligands, transmitting forces and signals of cell migration in physiological and pathological conditions. β1 integrin-deficient keratinocytes show impaired motility in vitro and a severe defect in wound healing in vivo (Grose et al., 2002). Full activation of β1 integrins in adherent cells requires both talin and kindlin-1 binding to the integrin cytoplasmic domain through the FERM domain, where W612 is essential for interaction and efficient cell adhesion (Harburger et al., 2009). Our experiments showed that β1 integrins are activated by EF in NHK but not KS cells, and a W612A mutation in kindlin-1 inhibited EF-induced activation, confirming that kindlin-1–integrin binding was required for EF-directed migration. β4 integrins have also been shown to be required for keratinocyte directional migration in EF (Pullar et al., 2006). The same study also reported that the cytoplasmic tail of β4 integrins is required for integrin-mediated directional migration of keratinocytes. However, β4 integrin shares very little sequence homology with β1 in the C-terminus and cannot associate with kindlins, suggesting that β4-mediated EF responses may be mediated through an alternate pathway.

Genetic disruption of PI(3)K decreases EF-induced directional migration in epithelial cells. Similarly, deleting phosphatase and tensin homolog in mice, human skin fibroblasts, and neuronal stem cells increases electrotactic responses (Guo et al., 2010, Meng et al., 2011, Zhao et al., 2006). However, how PI(3)K senses and transmits EF signals to and from the cytoskeleton and associated signaling responses remains unclear. The PH domain in kindlins is a phosphoinositide-binding site (Liu et al., 2011). Local synthesis of phosphoinositides, for example by PI(3)K, may be transiently increased at adhesion sites in the lamellipodia (Chen and Guan, 1994, Di Paolo et al., 2002, Yates et al., 2012). We report that PH domain-deleted kindlin-1 failed to restore electrotactic responses, suggesting that EF-induced PI(3)K signaling leading to polarized transient PIP3 production may then associate with the PH domain of kindlin-1 to stabilize leading edge protrusion. Integrin β1 function-blocking antibodies have also been reported to block PI(3)K/protein kinase A signaling pathway in fibroblasts (Tai et al., 2003). Therefore, activated integrins may also activate PI(3)K signaling in response to EF-induced gradients and form a positive-feedback loop to sustain persistent directed protrusion and focal adhesion dynamics.

In conclusion, we describe a role for kindlin-1 in sustaining directional migration of keratinocytes in response to EFs and show that the interaction of kindlin-1 with β1 integrins is important to maintain lamellipodia at the leading edge and achieve EF-mediated directional migration. Thus, kindlin-1 may act to relay local signals, via PI(3)K, to fully activate β1 integrins to stabilize the newly formed lamellipodia in the direction of the applied EF. Studying the function of kindlin-1 in electrotaxis will improve our understanding not only of skin integrity in KS patients but also the mechanisms of directional sensing and migration that are required for other biological processes such as embryonic development, inflammation, and tumor cell metastasis.

## Materials and Methods

### Antibodies and reagents

The following antibodies were used: anti-integrin β1 (12G10, Millipore, Billerica, MA) and anti-integrin β1 (P5D2, Abcam, Cambridge, UK). The following secondary antibodies were purchased from Invitrogen (Waltham, MA): Alexa Fluor 488 goat anti-mouse and Alexa Fluor 488 goat anti-rabbit.

### KS cell culture and generation

Immortalized normal human keratinocytes or those from a patient harboring known *FERMT1* mutations were used for this study (c.676insC/c.676insC (Martignago et al., 2007), and these cells are representative of behaviors seen in cells isolated from other KS patients (Lai-Cheong et al., 2009). The electrotaxis cell migration experiments were performed with 10–25 passages; no differences in biological responses between passage numbers were observed within this range. The study was conducted according to the principles of the Declaration of Helsinki. All cells were obtained under the St. Thomas Hospital Ethics Committee-approved project “Molecular basis of inherited skin disease—07/H0802/104.” Cells were maintained in KSFM media (Gibco, Waltham, MA), supplemented with epidermal growth factor and bovine pituitary extract. To generate stable cell lines of KS keratinocytes, wild-type, W612A, or deltaPH (amino acids 378–473) kindlin-1 was subcloned into pHR9SIN-SEW lentiviral expression vector containing an N-terminal mCherry tag (a gift from Adrian Thrasher, Institute of Child Health, University College London, London). Lentiviral DNA was transfected with the Δ8.91 packaging plasmid and pMD2.G envelope plasmid into HEK293 cells for lentiviral particle production. Virus was harvested and used to infect target keratinocytes. Expressing cells were further selected for expression using FACS and maintained for further passages under identical conditions to noninfected cells. Patient consent for experiments was not required because we used immortalized human cell lines, which had been subcultured more than 10 times.

### Cell migration in EFs and time-lapse imaging

The EF-induced cell migration experiment was performed according to previous publications (Song et al., 2007, Zhao et al., 2006). Briefly, direct current EFs in a physiological range of up to 200 mV/mm were applied through agar-salt bridges on either side of custom chambers maintained at 37 °C. Cell migration was recorded by DeltaVision imaging system (Imsol, Preston, UK) (Song et al., 2007). Mean directedness was calculated with the formula

∑ni=1cosθi=n,

where *n* is the total number of cells, and θ*i* is the angle between the vector of cell displacement and the EF vector. To classify the morphological behavior, cathodal protrusion was defined as occurring between 135° and 225° and anodal protrusion as between –45° and 45° with respect to the EF source, because these regions provide the most representative directions with respect to cathode or anode. Trajectory speed was calculated as the total distance traveled by the cells divided by the traveling time. Displacement speed was calculated as the straight-line distance between the start and endpoints of migrating cells divided by the traveling time. Transient cell-cell interactions were occasionally observed but did not affect the directional EF response under any cell densities tested.

### Flow cytometry

A total of 1 × 10^6^ keratinocytes were washed twice in phosphate buffered saline containing 0.3% bovine serum albumin. Primary and secondary antibody incubation was performed on live cells for 1 hour or 30 minutes, respectively. A parallel experiment using isotype controls was also conducted. FACS was performed using the BD Calibur flow cytometer (BD Biosciences, Oxford, UK).

### Immunocytochemistry

Cells were fixed with 4% paraformaldehyde for 10 minutes at room temperature and blocked with 5% fetal bovine serum (Invitrogen) in phosphate buffered saline, followed by primary antibody incubation overnight at 4 °C. The secondary antibody was incubated for 1 hour at 37 °C. The fixed coverslips were analyzed with a DeltaVision Imaging system (Imsol). Analysis of intensity of integrin staining was performed in ImageJ (National Institutes of Health, Bethesda, MD).

### Analysis of the pseudopod dynamics

Pseudopod dynamics analysis was conducted with the Quimp3 algorithm following a previous description (Bosgraaf and Van Haastert, 2010a). Briefly, the recorded cell migration movie was tracked using ImageJ software (National Institutes of Health) with the pseudopod macro. The movements of each pseudopod, including genesis, split, maintain, or disappear, and the angles of cell movement in relation to the EF vector were calculated.

## Conflict of Interest

The authors state no conflict of interest.

## Acknowledgments

This study was funded by European Research Council StG grant 243261 and the British Council Global Innovation Initiative Award (to BS), the British Skin Foundation and the UK National Institute for Health Research Biomedical Research Centre based at Guy’s and St Thomas’ National Health Service Foundation Trust and King’s College London (MP and JAM), and Grants of Innovative Research Teams of Liaoning Province, China (LT2011012 to XHG). The views expressed are those of the authors and not necessarily those of the National Health Service, the National Institute for Health Research, or the UK Department of Health.

## Author Contributions

BS, MP & GZ designed the study and wrote the paper. GZ, YG & RB conducted the experiments and data analysis. HDC, XHG & JAM contributed to the data analysis, discussion and paper writing. BS led the study regarding electric stimulation and cell migration analysis. MP created KS and NHK cell lines and constructed plasmids. BS, XHG and MP provided financial support to the project.
