# Supplementary material for: Kindlin-1 Regulates Keratinocyte Electrotaxis
Source: J Invest Dermatol. 2016 Nov;136(11):2229–39. doi: 10.1016/j.jid.2016.05.129 (PMC5756539; doi:10.1016/j.jid.2016.05.129)
Supplement: Supplementary Figure S1 [file mmc1.pdf]

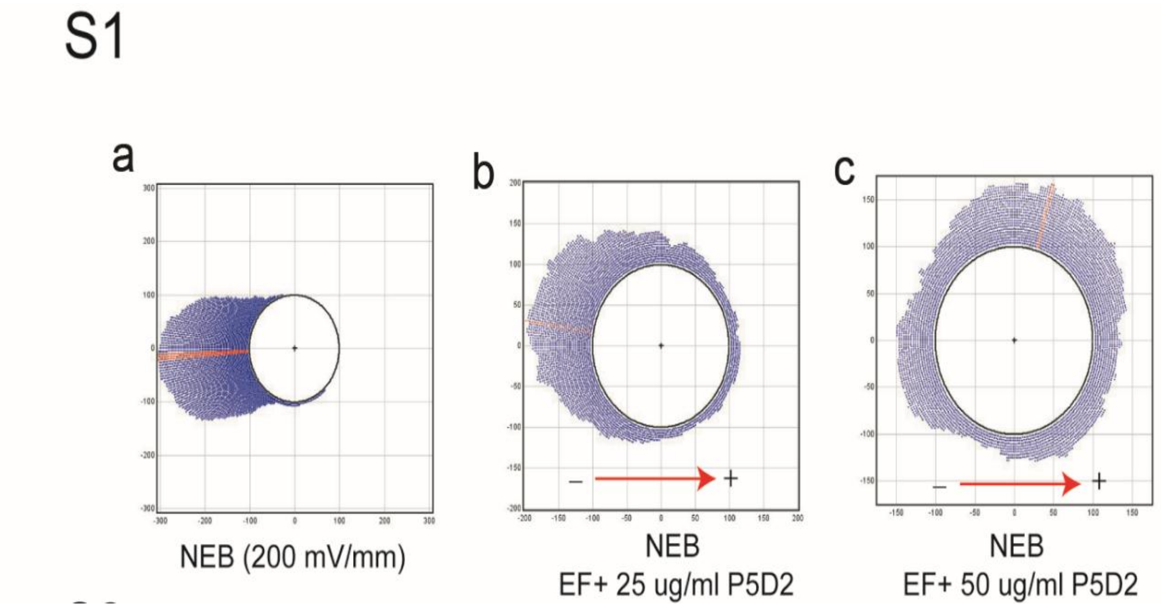

Supplementary Figure S1. (a-c) The electrotaxis of NHK cells with control IgG (a), 25 µg/ml (b) and 50µg/ml (c) were analysed by using circular graphs. x and y-axis give distance in µm. EF vector is horizontal with cathode to the left.
